# Sex‐dependent Biomarker Signatures in Alzheimer's Disease

**DOI:** 10.1002/alz.092885

**Published:** 2025-01-09

**Authors:** Lavanya Jain, Maria Khrestian, Elizabeth D. Tuason, Jagan A. Pillai, Stephen M. Rao, James B Leverenz, Lynn M. Bekris

**Affiliations:** ^1^ Cleveland Clinic Genomic Medicine Institute, Cleveland, OH USA; ^2^ Cleveland Clinic Lou Ruvo Center for Brain Health, Cleveland, OH USA

## Abstract

**Background:**

Alzheimer’s disease (AD) is a pathologically heterogeneous disease making it a challenge to develop effective treatments. Emerging evidence suggests that there are pathological differences between women and men with AD. More biomarkers are needed to enhance sex‐specific precision medicine in AD. Cerebrospinal fluid (CSF) biomarkers for amyloid (A), tau (T), and neurodegeneration (N) can be utilized to more precisely define pre‐mortem pathological status (ATN research framework) to limit the heterogeneity of a study cohort. The hypothesis of this study was that non‐ATN biomarkers differ by sex and ATN in AD. The overall aim of this preliminary investigation was to understand sex differences in seven biomarkers in an ATN defined AD cohort.

**Methods:**

Cerebrospinal fluid (CSF) samples from an AD/MCI cohort were obtained from the Cleveland Alzheimer’s Disease Research Center and Cleveland Clinic Lou Ruvo Center for Brain Health Aging and Neurodegenerative Disease Biobank. CSF Aβ_42/40_ (A), p‐Tau181 (T), and t‐Tau (N), were evaluated for ATN analysis. Only A‐T‐N‐ individuals were included in CN and only A+T‐N‐, A+T+N‐, and A+T+N+ were included in as MCI and AD (MCI/AD) individuals. Seven biomarkers namely, neurofilament light chain (NFL), soluble Triggering receptor expressed on myeloid cells 2 (sTREM2), Neurogranin, fatty acid binding protein 3 (FABP3), glial fibrillary acidic protein (GFAP), neuron specific enolase (NSE), and α‐synuclein (α‐syn) (were also evaluated. Statistical analyses such as ROC analysis and one‐way ANOVA were carried out in RStudio.

**Results:**

This was a cohort of over 300 individuals out of which approximately 50% were females and 50% were males. Males exhibited differences between the CN (A‐T‐N‐) and MCI/AD (A+T‐N‐, A+T+N‐, A+T+N+) group for NFL, Neurogranin, FABP3, NSE, and α‐syn, whereas females showed for NFL, sTREM2, and α‐syn. When comparing ATN status between sexes, GFAP was found to be higher in CN (A‐T‐N‐) males and MCI/AD A+T+N+ males, and sTREM2 was higher in MCI/AD A+T+N‐ males.

**Conclusions:**

These preliminary results show significant differences between and within sex for these selected seven biomarkers. With validation from a larger dataset and more robust statistical analyses, this could have crucial impact on AD related diagnostic and therapeutic approaches.

